# *Syzygium cumini* (L.) Extract-Derived Green Titanium Dioxide Nanoparticles Induce Caspase-Dependent Apoptosis in Hepatic Cancer Cells

**DOI:** 10.3390/plants12183174

**Published:** 2023-09-05

**Authors:** Musaed Rayzah, Abozer Y. Elderdery, Nasser A. N. Alzerwi, Badr Alzahrani, Abdullah Alsrhani, Afnan Alsultan, Bandar Idrees, Fares Rayzah, Yaser Bakhsh, Ahmed M. Alzahrani, Suresh K. Subbiah, Pooi Ling Mok

**Affiliations:** 1 Department of Surgery, College of Medicine, Majmaah University, Al Majmaah 11952, Saudi Arabia; 2 Department of Clinical Laboratory Sciences, College of Applied Medical Sciences, Jouf University, Sakaka 42421, Saudi Arabia; 3 Department of Surgery, King Saud Medical City, Riyadh 12746, Saudi Arabia; 4 Department of Surgery, Prince Sultan Military Medical City, As Sulimaniyah 12233, Saudi Arabia; 5 Aseer Central Hospital, Abha 62523, Saudi Arabia; 6 Iman General Hospital, Riyadh 12211, Saudi Arabia; 7 Centre for Materials Engineering and Regenerative Medicine, Bharath Institute of Higher Education and Research, Chennai 600073, India; 8 Department of Biomedical Science, Faculty of Medicine & Health Sciences, Universiti Putra Malaysia, UPM, Serdang 43400, Malaysia

**Keywords:** *Syzygium cumini*, titanium oxide, nanomedicine, liver cancer, apoptosis

## Abstract

An aqueous extract of *Syzygium cumini* seeds was utilized to green synthesize titanium dioxide nanoparticles (TiO_2_ NPs). UV-Visible, DLS, FTIR, XRD, FESEM, TEM, SAED, EDAX, and photoluminescence spectroscopy techniques were employed to characterize the prepared TiO_2_ nanoparticles. The rutile crystal structure of TiO_2_ NPs was revealed by XRD study. The TEM and FESEM images of the TiO_2_ NPs revealed an average particle size of 50–100 nm. We employed EDAX to investigate the elemental compositions of TiO_2_ NPs. The O-Ti-O stretching bands appeared in the FTIR spectrum of TiO_2_ NPs at wavenumbers of 495 cm^−1^. The absorption edge peaks of TiO_2_ NPs were found in the UV-vis spectra at 397 nm. The MTT study revealed that TiO_2_ NPs effectively inhibited the growth of liver cancer Hep3 and Hep-G2 cells. The results of the corresponding fluorescent staining assays showed that TiO_2_ NPs significantly increased ROS generation, decreased MMP, and induced apoptosis in both liver cancer Hep3 and Hep-G2 cells. TiO_2_ nanoparticles lessened SOD, CAT, and GSH levels while augmenting MDA contents in Hep3 and Hep-G2 cells. In both Hep3 and Hep-G2 cells treated with TiO_2_ NPs, the Bax, CytC, p53, caspase-3, -8, and -9 expressions were remarkably augmented, while Bcl-2 expression was reduced. Overall, these findings revealed that formulated TiO_2_ NPs treatment considerably inhibited growth and triggered apoptosis in Hep3 and HepG2 cells.

## 1. Introduction

Green synthetic nanoparticles have been proposed as potential frontiers for the development of nanotechnology due to being less hazardous to humans and ecosystems. Green synthesis increasingly employs the utilization of biological agents, particularly plant extracts, in place of hazardous chemical agents [1]. Green synthesis has many benefits, including low production costs and a simple one-step process [2]. Green synthetic nanoparticles have previously made use of several plant extracts due to their convenience in production [3].

Cancer is a collection of disorders characterized by uncontrolled, unchecked cell proliferation that leads to aberrant tissue growth, collectively known as malignant neoplasm [4]. Liver cancer is the fifth most frequent cancer and the third biggest reason of cancer-related fatalities globally, accounting for over 800,000 deaths annually [5]. If untreated, patients with liver cancer have a median survival time of 1–3 months. The most promising methods for treating liver cancer are surgery, chemotherapy, and radiation therapy. Though, only about 15% of individuals are reportedly suitable for surgery. The other 85% of patients with poor prognoses, metastasis, or abnormal liver function (such as underlying cirrhosis) are directed to alternative treatments [6]. As a result, there has been a rise in the need for novel treatment approaches that are both successful and efficient at resolving these problems.

Multidrug-resistant (MDR) pathogens are a foremost public health problem because they are resistant to currently available drugs, resulting in poor therapy, disease persistence, and transmission [7]. Over the past decade, a huge amount of money has been invested in nanomaterials, with an emphasis on drug delivery systems, because of their precise targeting mechanisms, increased efficacy, and reduced adverse effects [8]. The interaction of metal oxide NPs with microbial cells and other biological systems is significantly influenced by nano-size particles; because of their larger surface-to-volume ratios and surface defects (oxygen), metal oxide (MO) NPs exhibit better antimicrobial activity than bulk particles. Reactive oxygen species (ROS) are significant antimicrobial efficacy parameters. The antimicrobial mechanism of MO NPs is the excessive production of ROS in cells, thereby facilitating cell necrosis. MO NPs (such as TiO_2_, ZnO, Fe_2_O_3_, and MgO) are photocatalytic semiconductors that can generate ROS on their particle surfaces when exposed to light [9].

Titanium dioxide (TiO_2_) nanoparticles have gained much interest in recent times because of their distinctive properties and potential utilizations in numerous areas, including medicine. In the context of anticancer activity, TiO_2_ NPs was studied for their capability as a drug delivery system and therapeutic agent [10]. TiO_2_ nanoparticles can be utilized as carriers for anticancer drugs, and their small size and high surface area that makes them suitable for encapsulating and delivering drugs to tumor sites. Researchers have explored different strategies to load anticancer drugs onto TiO_2_ nanoparticles, such as physical adsorption, covalent bonding, and electrostatic interactions. By functionalizing the surface of TiO_2_ NPs, it is able to enhance their targeting efficiency and improve drug release kinetics. This allows for controlled and targeted drug delivery [11].

TiO_2_ nanoparticles exhibit photocatalytic properties that can be harnessed for photodynamic therapy. In photodynamic therapy (PDT), TiO_2_ nanoparticles are excited by light of a specified wavelength, resulting in the production of ROS that specifically kill tumor cells. When TiO_2_ nanoparticles are exposed to light, they produce singlet oxygen and other ROS, which induce cell death through oxidative stress [12]. This approach has shown promise in preclinical studies and is minimally invasive and highly localized. Apart from their drug delivery and PDT applications, TiO_2_ NPs themselves have demonstrated potential anticancer properties [13].

Works have revealed that TiO_2_ NPs trigger cytotoxicity and inhibit cancer cell growth through multiple mechanisms. These mechanisms include the generation of ROS, disruption of cellular signaling pathways, interference with DNA repair mechanisms, and induction of apoptosis in tumor cells [14]. However, it is imperative to note that the effectiveness of TiO_2_ nanoparticles as an anticancer agent may depend on several causes, including nanoparticle size, surface functionalization, and the specific type of cancer being targeted. It is worth mentioning that while TiO_2_ nanoparticles hold promise for anticancer applications, further study is required to completely comprehend their safety profile, optimize their therapeutic efficacy, and overcome potential challenges associated with their use in clinical settings [15].

The green production of TiO_2_ NPs has received the curiosity of several researchers because of the high cost of their chemical and physical processes and the extreme reaction conditions. Therefore, plant extracts have been used by researchers in their exploration for novel low-cost routes for NPs synthesis. *Syzygium cumini* (*S. cumini*) is a popular medicinal plant that possesses antioxidant and anti-inflammatory properties [16]. In the current exploration, the TiO_2_ NPs were formulated by a green synthesis process using an *S. cumini* seed extract. The formulated TiO_2_ NPs were studied for their structural, morphological, optical, and antimicrobial properties.

## 2. Results and Discussion

Several previous studies have already highlighted the production of TiO_2_ NPs using plant extracts, including jasmine flower [17], lemon peel extract [18], citrus limon juice extract [18], *Eichhornia crassipes* extract [19], *Olea europaea* extract [20], lemon peel extract [21], cinnamon powder extract [22], orange peel extract [23], *Jatropha curcas* extract [24], and pomegranate peel extract [25]. In the current work, the TiO_2_ nanoparticles are synthesized via a green process using *Syzygium cumini* seed extract to avoid using hazardous (toxic) organic solvents, surfactants, and capping agents often used in chemical synthesis. Among them, plant-based material fabrication is reproducible, and the green synthesized nanomaterials are hydrophilic (strong affinity for water). For example, during the formation of TiO_2_ NPs, when 0.1 M of aqueous titanium isopropoxide solute was mixed with the *Syzygium cumini* seed extract solution, the homogenous Ti metal ion’s reaction mixture turned light green with a colorless white precipitate. In addition, the plant extract contains phytocomponents such as amino groups and protein groups that play crucial roles in the reduction of the Ti^4+^ ions to form the TiO_2_ NPs.

### 2.1. Characterization of TiO_2_ NPs

**Figure 1:**
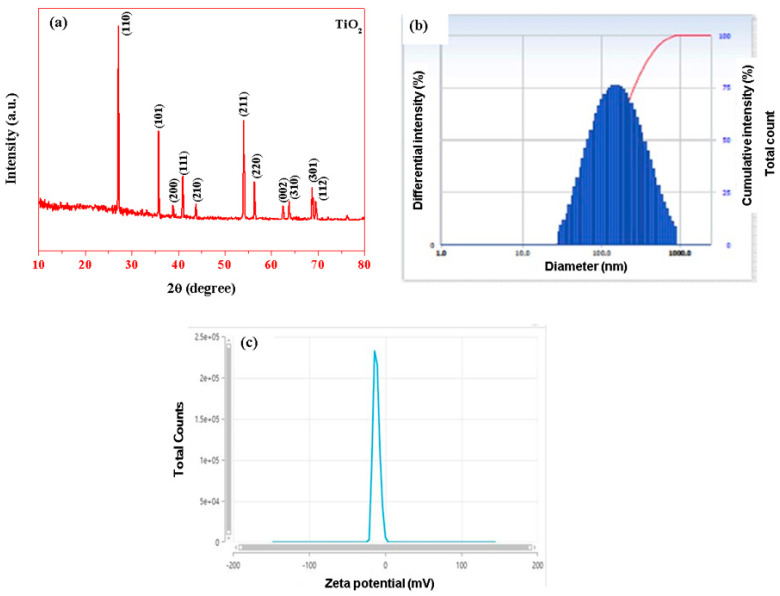
XRD analysis (**a**), DLS spectrum (**b**), and Zeta potential (**c**) of TiO_2_ NPs. The representative images were obtained from the triplicate experiments.

The XRD pattern of the highly crystalline green synthesis of TiO_2_ NPs is demonstrated in Figure 1a. The TiO_2_ NPs were found to be well crystalline titanium dioxide rutile phase (JCPDS card No: 21-1276) based on XRD results [26]. The diffraction peaks of TiO_2_ NPs in the rutile phase of 2θ angle were at 27.16, 35.79, 38.92, 40.97, 43.81, 54.05, 56.35, 62.45, 63.78, 68.72, and 69.50⁰, corresponding diffraction plans (110), (101), (200), (111), (210), (211), (220), (002), (310), (301), and (112), respectively. *Pouteria campechiana* leaf extract mediated to TiO_2_ NPs exhibits a rutile phase in the early literature [27]. The average particle size was calculated by the Debye Scherrer equation on the rutile diffraction peaks [28]. The average crystallite size D = $\frac{k\lambda }{\beta_{Dcos\theta }}$. The average particle size was observed at 55 nm for TiO_2_ NPs. The hydrodynamic size of TiO_2_ NPs observed at 120 nm was used to measure the DLS spectrum (Figure 1b,c). The Polydispersity index (PI) was at 0.47 and the Zeta potential at −11.73 mV.

### 2.2. Morphology Analysis and Chemical Composition

**Figure 2:**
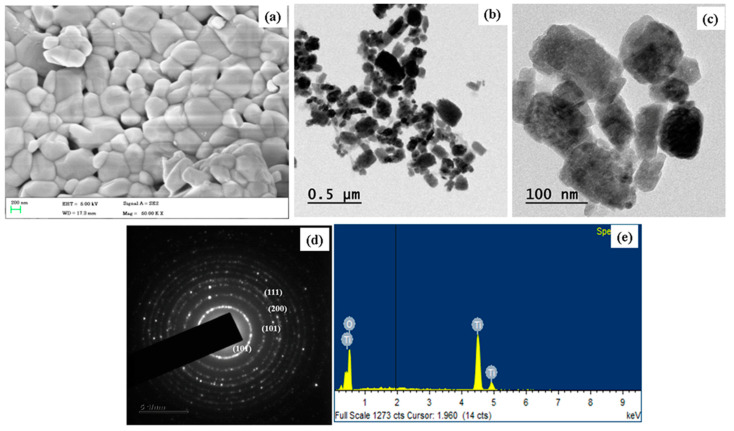
FESEM (**a**), TEM (**b**,**c**), and SAED (**d**) pattern images of TiO_2_ NPs and EDAX spectrum (**e**).

The formulated TiO_2_ NP’s morphology was studied using FE-SEM and TEM analysis, as revealed in Figure 2a,b. The TiO_2_ NPs are formed using titanium isopropoxide and *S. cumini* seed extract. The FE-SEM and TEM microphotographs of the TiO_2_ NPs showed a river stone-like morphology (polygonal). The average size of the TiO_2_ NPs was 50–100 nm. The SAED pattern prepared TiO_2_ NPs (Figure 2c) that can be assigned to the reflections (110), (101), (200), (111), (210), (211), (220), (002), (310), (301), and (112) of the rutile phase of TiO_2_ NPs. There are no additional rings in the SAED pattern caused by crystalline impurities. The elemental configuration of TiO_2_ NPs was investigated via an EDAX spectrum. Figure 2d shows the EDAX spectrum of TiO_2_ NPs. The atomic percentage of TiO_2_ NPs was Titanium (Ti) at 23.33% and Oxygen (O) at 76.67%, respectively.

**Figure 3:**
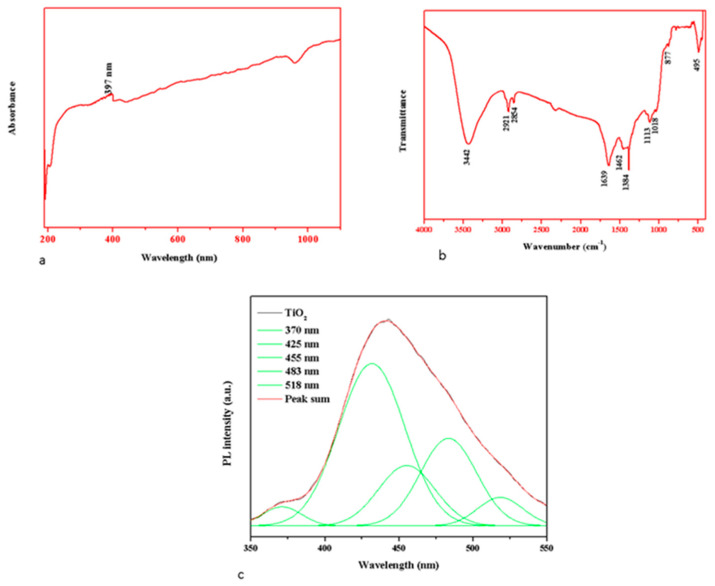
An analysis of TiO_2_ NPs by UV absorbance (**a**), FTIR spectrum (**b**), and PL spectrum (**c**). The representative images were obtained from the triplicate experiments.

The optical properties depend on numerous factors, including the band gap, oxygen deficiency, and surface roughness. The UV-Vis absorbance spectrum of TiO_2_ NPs is revealed in Figure 3a. During the green synthesis, the colloidal suspension turns from white to yellowish-green, corresponding to the TiO_2_ NPs development. The UV absorbance peak around the 385–400 nm area confirmed the development of TiO_2_ NPs [29]. The present work observed the absorbance edge at 397 nm for TiO_2_ NPs, indicating that the transition of charge coordinated electron between the O 2p and Ti 3d states [30]. The hydrodynamic diameter of green synthesized TiO_2_ NPs averaged 156 nm; because a water medium surrounded the TiO_2_ NPs, the particle size was increased (Figure 3b) compared with the XRD outcomes. This is known as hydrodynamic size [31].

The FTIR spectra of formulated TiO_2_ NPs is revealed in Figure 3b. The biomolecules of *S. cumini* functional groups, such as polyphenolic group O-H stretching vibrations were detected at 3442 cm^−1^, asymmetric and symmetric C–H groups located at 2921 to 2854 cm^−1^, O-H observed at 1462 cm^1−1^, while the O-Ti-O bond and the metal–oxygen bond (Ti-O) of TiO_2_ NPs formation peaks were observed at 495 cm^−1^ [32,33].

The photoluminescence spectrum of TiO_2_ NPs has an excitation wavelength of 425 nm, as depicted in Figure 3c. The PL emission spectrum of TiO_2_ NPs was found at 370 nm, 425 nm, 455 nm, 483 nm, and 518 nm, respectively. The UV emissions were noted at 370 nm because of the free exciton–exciton collision’s radiative recombination. The transfer of electron from the surface donor of the tin interstitials (Ti_i_) to the top of the valence band was responsible for the violet emission detected at 425 nm. Singly ionized tin vacancies (V_Ti_) were exhibited by the blue emissions at 455 nm and 483 nm, respectively. The green emission band was centred at 530 nm because of oxygen vacancies (O_v_) [34].

### 2.3. Cell Viability Assay

Cancer cells have an impressive capacity for self-replication and metastasis. Unchecked and uncontrolled cell growth leads to the formation of malignant tissues, which are prone to metastasizing to other body areas [35]. The main issue with cancer treatments is their low therapeutic index and lower ability to distinguish between malignant cells and normal cells. The efficiency and safety of treatment are limited by anticancer agents with low selectivity, which kill both tumor and non-malignant cells [36]. The life quality of cancer survivors is severely reduced by the numerous negative side effects associated with these strategies. To rectify these problems and improve the efficacy of cancer treatment, novel nanomedicines are being developed [37]. In this work, to prove the anticancer properties of the formulated TiO_2_ NPs, we used the MTT assay to assess if TiO_2_ NPs might inhibit the growth of liver cancer Hep3 and Hep-G2 cells.

**Figure 4:**
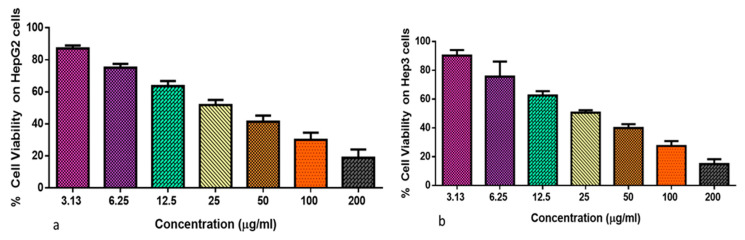
Cytotoxic effects of TiO_2_ NPs against Hep-G2 (**a**) and Hep3 (**b**) cells. The diverse doses (3.13, 6.25, 12.5, 25, 50, 100, and 200 µg/mL) of TiO_2_ NPs were treated to Hep-G2 (**a**) and Hep3 (**b**) cells. Assays were carried out in triplicate to calculate the IC_50_ dose, which resulted in 50% growth inhibition.

Figure 4a,b displays the results of an analysis into the cytotoxicity of formulated TiO_2_ NPs on both Hep3 and Hep-G2 cells. Our outcomes revealed that the growth of Hep3 and Hep-G2 cells was drastically lowered upon treatment with TiO_2_ NPs at varying concentrations (3.13–200 µg). These findings show that TiO_2_ NPs are cytotoxic to liver cancer cells (Figure 4b). Given that 29.43 and 27.11 µg/mL of TiO_2_ NPs was shown to inhibit 50% of the viability in HepG2 and Hep3 cells, respectively, hence 29.43 and 27.11 µg/mL were selected as the IC_50_ (high dose) and 14.7 and 13.5 µg/mL were selected as the IC_25_ (Low dose) for the remaining assays.

### 2.4. Apoptosis Analysis by Dual Staining

Defective apoptosis, in addition to unchecked cell proliferation, is a significant contributor to tumor formation. Apoptosis serves as a protective mechanism against the growth of malignancies. When neoplastic cells develop a resistance to apoptosis due to genetic and epigenetic alterations, the phenomenon is known as oncogenic transformation. Defective apoptosis regulation in carcinogenesis results in extended tumor cell survival, stress-induced proliferation, and metastasis. Additionally, it increases the tumor cell’s resistance to therapy [38]. Therefore, the examination of the ability of novel anticancer candidates to trigger apoptosis in cancer cells is necessary.

**Figure 5:**
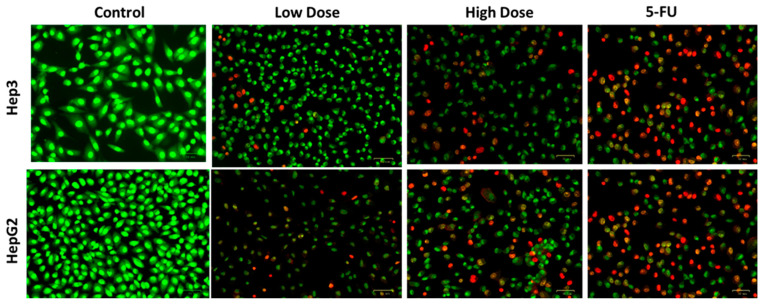
Apoptosis was examined in Hep3 and HepG2 cells by AO/EtBr staining with IC_50_ of TiO_2_ NPs after 24 h of treatment. Green colored cells indicate viable cells, cells with yellowish red indicate early apoptosis, and cells with red color indicate late apoptosis. Control cells, cells were treated with low dosage (IC_25_), high dosage (IC_50_) of TiO_2_ NPs, and 5-FU as the positive control drug. Assays were executed in triplicate and microphotographs were captured at 20× magnification.

Figure 5 shows the results of dual staining on the TiO_2_ NP’s impact on apoptosis in Hep3 and Hep-G2 cells. In comparison to control cells, Hep3 and Hep-G2 cells that were subjected to IC_50_ concentration of TiO_2_ NPs exhibited a strong orange/yellow fluorescence along with altered cell shape, rounding, and nuclear damage, which was also seen in the 5-FU treated cells. Therefore, it was clear that TiO_2_ NPs administration promoted early and late apoptosis in the Hep3 and Hep-G2 cells.

### 2.5. Analysis of Nuclear Damage by Comet Assay

Apoptosis has a complex pathophysiology that includes both intrinsic and extrinsic signal transmission [39]. Numerous mechanisms, such as the overexpression of anti-apoptotic genes, are produced by cancer cells to inhibit apoptotic cell death. DNA damage in tumor can lead to apoptosis, which kills possibly dangerous cells and stops the growth of tumors. On the other hand, apoptotic malfunction can result in unchecked cell proliferation, tumor development, and therapy-resistant tumors [40]. In order to stop the growth of tumors, it is thought that inducing apoptosis in cancer cells is the most effective method.

**Figure 6:**
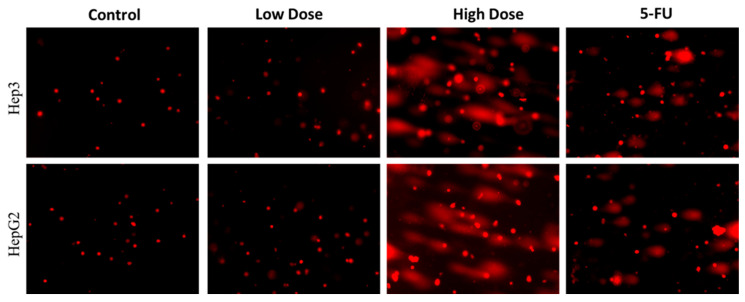
Comet assay for TiO_2_ NPs treated Hep3 and HepG2 cells showing detectable comet tails, indicative of DNA damage. Control cells, cells treated with low dosage (IC_25_), high dosage (IC_50_) of TiO_2_ NPs, and 5-FU as positive control drug. Assays were executed in triplicate and microphotographs were captured at 20× magnification.

The apoptotic cell nuclear damage in the cells were assessed by the comet assay. Figure 6 reveals that the control cells did not develop tails, indicating that they do not have nuclear damage. Contrarily, Hep3 and Hep-G2 cells subjected to IC_50_ concentration of formulated TiO_2_ NPs show a clear tail development, suggesting that the TiO_2_ NPs enhanced nuclear damage in the Hep3 and Hep-G2 cells. The 5-FU treated cells also developed a tail formation, which proves the nuclear damage.

### 2.6. Apoptosis Analysis by DAPI Staining

Apoptosis is characterized by morphological changes, some of which can serve as early signs of impending cell death. Overall, apoptosis begins with chromatin condensation, fragmentation of nucleus, and the development of apoptotic bodies [40]. Resistance to apoptosis commonly occurs when cell death or pro-survival signaling is dysregulated during tumor development [41].

**Figure 7:**
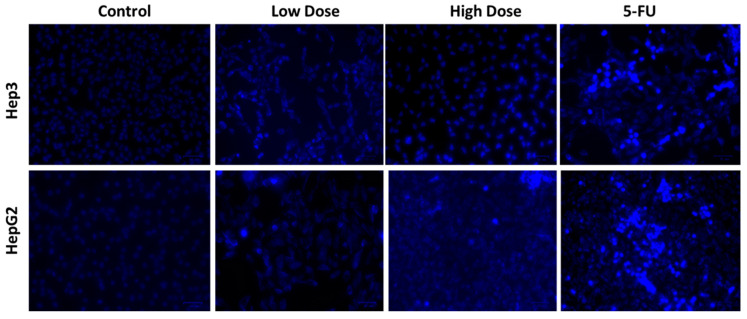
DAPI staining was employed to measure apoptosis in Hep3 and HepG2 cells after 24 h of exposure to TiO_2_ NPs. Control cells, cells treated with low dosage (IC_25_), high dosage (IC_50_) of TiO_2_ NPs, and 5-FU as positive control drug. Assays were executed in triplicate and microphotographs were captured at 20× magnification.

Figure 7 shows the results of DAPI staining to assess the impacts of TiO_2_ NPs on the nuclear morphology of apoptotic cells in Hep3 and Hep-G2 cells. DAPI staining results exhibited that TiO_2_ NPs treatment increased apoptosis in Hep3 and Hep-G2 cells, as the cells exposed to IC_50_ of TiO_2_ NPs exhibited clear alterations in nuclear morphology, including condensation, shrinkage and the development of apoptotic bodies (Figure 7).

### 2.7. MMP Analysis by Rh-123 Staining

**Figure 8:**
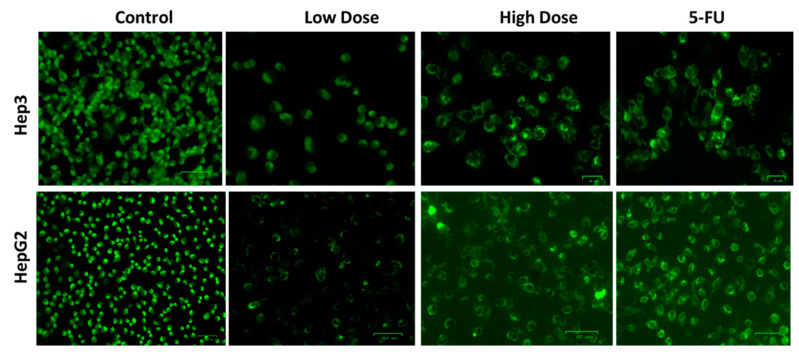
In Hep3 and HepG2 cells treated for 24 h with TiO_2_ NPs, the MMP was measured by Rh-123 staining. Control cells, cells exposed to low dosage (IC_25_), high dosage (IC_50_) of TiO_2_ NPs, and 5-FU as positive control drug. Assays were executed in triplicate and microphotographs were captured at 20× magnification.

An earlier report by He et al. [42] revealed that the depletion of the MMP is a pivotal cause of inducing apoptosis. Figure 8 displays the MMP level of untreated and TiO_2_ NPs treated liver cancer cells, analyzed by Rh-123 staining. The control cells demonstrated unchanged and intact MMP, as seen by the intense green fluorescence. However, low green fluorescence indicated that MMP status was decreased in Hep3 and Hep-G2 cells exposed to the IC_50_ level of TiO_2_ NPs (Figure 8). The standard drug 5-FU treatment also revealed decreased green fluorescence, which proves the reduction in MMP level.

### 2.8. Analysis of Oxidative Stress Markers

In healthy cells, redox balance is maintained by tight control over free radical production and antioxidant defense. The GSH, SOD, and CAT protect cells from damage caused by ROS and other free radicals. Antioxidant enzymes such as SOD and CAT are essential for protecting cells from free radical damage. When it comes to preventing lipid peroxidation, GSH is essential [43]. Cancer cells’ ability to create antioxidants can assist in removing excessive ROS, which in turn allows them to avoid apoptosis and increase their chances of survival. Conversely, increased oxidative stress in cancer cells might activate anti-tumorigenic signaling cascades, leading to tumor cell necrosis [44].

**Figure 9:**
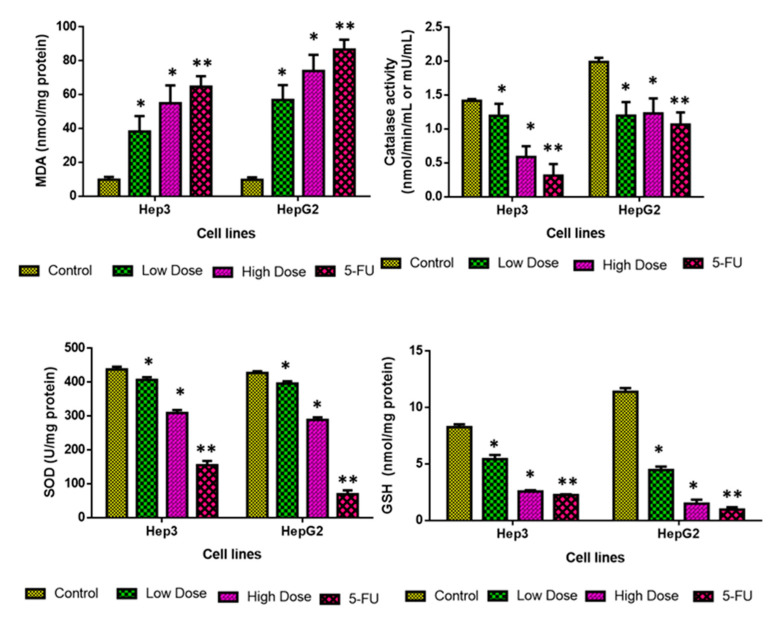
Effects of TiO_2_ NPs on oxidative stress markers in Hep3 and HepG2 cells. CAT activity (one unit) is specified as a level of protein that degrades 1 μmol H_2_O_2_/s. SOD activity (one unit) is specified as a protein level needed to inhibit 50% of the SOD activity, where superoxide radicals oxidize hydroxylamine to form nitrite. Values depicted as mean ± SD of the triplicate. Each bar of treatment groups significantly differed at * *p* < 0.05, ** *p* < 0.001 when compared to control.

The influence of TiO_2_ NPs treatment on the MDA, CAT, GSH, and SOD levels in the Hep3 and Hep-G2 cells are depicted in Figure 9. The level of MDA considerably increased in both Hep3 and Hep-G2 cells, which were treated with the IC_25_ concentration of TiO_2_ NPs. Furthermore, the GSH, CAT, and SOD levels were effectively decreased by the IC_50_ Concentration of TiO_2_ NPs. The 5-FU treatment also increased the MDA level while decreasing the CAT, SOD, and GSH levels in the Hep3 and Hep-G2 cells, which supports the activity of TiO_2_ NPs (Figure 9). These findings proved that TiO_2_ NPs treatment decreases antioxidants while elevating the MDA level, thereby facilitating oxidative stress-regulated cell death in Hep3 and Hep-G2 cells.

### 2.9. Analysis of Apoptotic Protein Levels

Cancer cells frequently undergo a complex series of molecular processes to escape apoptosis and acquire resistance to apoptosis. Apoptosis is highly controlled by Bcl-2 family protein members, including pro- and anti-apoptotic genes [45]. In order to suppress Bax expression and avoid apoptosis, tumor cells commonly increase Bcl-2 protein expression.

**Figure 10:**
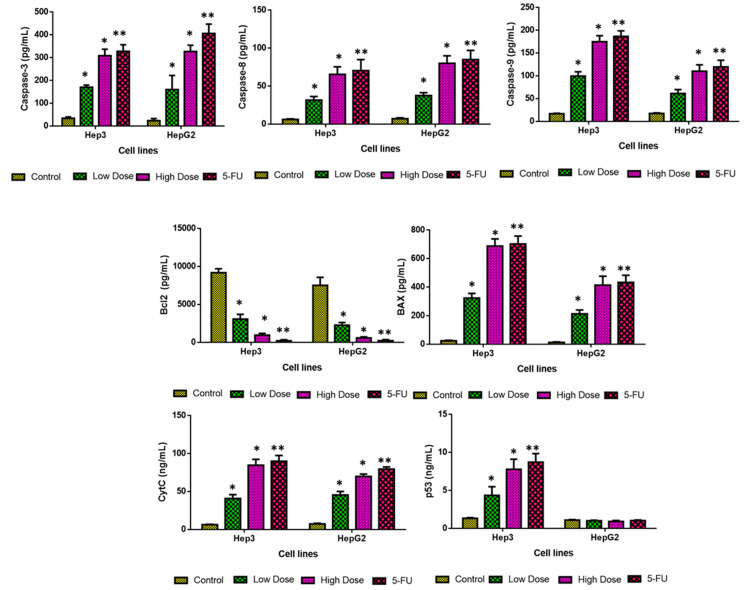
TiO_2_ NPs inhibits cell proliferation and promotes apoptosis in Hep3 and HepG2 cells. Caspase-3, 8, 9, Bax, Bcl-2, CytC, and p53 in both cells were studied using corresponding ELISA kits. All the assays were carried out in triplicate and the outcomes are depicted as mean ± SD. Multiple T-test were carried out to investigate the data. * *p* < 0.05, ** *p* < 0.001 indicates the *p* value of test when compared to the control.

Figure 10 shows the results of an analysis of the Bcl-2, Bax, CytC, p53, and caspase-3, -8, and -9 expressions in the control and treated cells. Treatment of both Hep3 and Hep-G2 cells with an IC_50_ concentration of TiO_2_ NPs resulted in the augmented expression of Bax, CytC, p53, and caspase-3, -8, and -9. Contrarily, treatment with TiO_2_ NPs reduced Bcl-2 expression in both Hep3 and Hep-G2 cells, indicating that TiO_2_ NPs treatment enhances apoptotic protein expressions in the liver cancer cells (Figure 10). The 5-FU treatment also increases the apoptotic protein expressions in the liver cancer cells, which corroborates the TiO_2_ NPs activity.

In more resistant malignancies, inactivation or downregulation of pro-apoptotic genes contributes to the tumor’s resistance to treatment [46]. Bax was showed to block Bcl-2 expression, leading to a change in MMP. According to the literature, Bcl-2 inhibits apoptotic cascade activation, which suggests it may be a useful therapeutic target for cancer treatment [47]. By modulating the pro- and anti-apoptotic protein expressions, caspase activation is a major cause of apoptosis [48]. Overall, our findings demonstrated that TiO_2_ NPs treatment considerably boosted the Bax, CytC, p53, and caspase-3, -8, and -9 expressions, thereby facilitating the apoptosis in Hep3 and Hep-G2 cells.

As a result, this study demonstrates that titanium dioxide nanoparticles derived from *Syzygium cumini* (L.) seed extract can induce caspase-dependent apoptosis of hepatic cancer cells, which is promising for the treatment of this disease. There is a need for further research into these issues, including clinical data, bioavailability, and in vivo studies, so that these findings can be translated into effective therapies for humans.

## 3. Materials and Methods 

### 3.1. Syzygium Cumini Seed Extract Preparation

The fruit pericarp was taken out from the seeds after collecting *S. cumini* fruits from the Gandhi market in Tiruchirappalli, Tamil Nadu, India. The separated seeds were washed several times in deionized water to discard the fruit fleshes. *S. cumini* seeds were then cut into pieces and dehydrated for one month at 37 °C. The dehydrated samples were powdered using a mechanical grinder. In a 250 mL beaker, 10 g of *Syzygium cumini* seed powder was boiled with 100 mL of deionized water and agitated with a magnetic stirrer for about an hour at 80 °C. The aqueous solution’s color changed from watery to a light yellowish brown. The solution was cooled to 37 °C, filtered and stored at −4 °C until future use.

**Figure 11:**
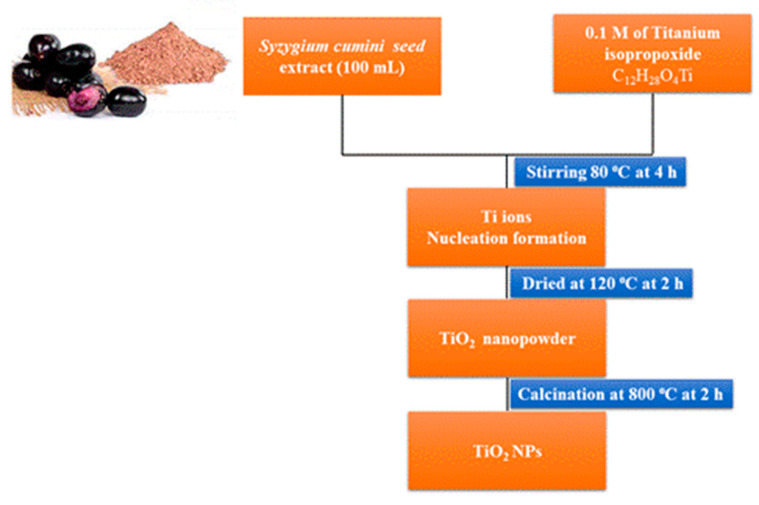
Formation of the TiO_2_ NPs from *Syzygium cumini* seed extract.

At room temperature (RT), 100 mL of *S. cumini* seed aqueous extract was mixed with 0.1 M titanium isopropoxide solute. The homogenous reaction mixture was heated at 80 °C for 4 h. The resulting suspension was permitted to cool at 37 °C and centrifuged for 15 min at 8000 rpm. The nanopowder was dehydrated at 120 °C for 2 h. The obtained TiO_2_ NPs were calcined at 800 °C in the atmosphere for 2 h and were used for further characterization. The formation of TiO_2_ NPs flow chart is given in Figure 11.

### 3.2. Characterization Techniques

The XRD analysis was undertaken using an X-ray meter (X’PERT PRO Analytical). The diffraction patterns for TiO_2_ NPs were recorded in the 20–80° range using a monochromatic wavelength of 1.54 Å. The FE-SEM (Carl Zeiss Ultra-55 FESEM, Carl Zeiss AG, Oberkochen, Germany) with EDX (model: Inca) was utilized to study the TiO_2_ NPs. The appearance of the TiO_2_ NPs were studied using the TEM (Tecnai-F20, FEI Company, Hillsboro, OR, USA) operated at an accelerating voltage of 200 kV. The size of the particles was detected by the DLS using the Nano-Plus machine. The FT-IR spectrum was studied using a Perkin-Elmer machine at 400–4000 cm^−1^. At 37 °C, a luminescence spectrophotometer (Perkin-Elmer LS-5513, PerkinElmer, Hong Kong) and a xenon lamp was employed to detect photoluminescence.

### 3.3. TiO_2_ NPs Dispersion

TiO_2_ NPs was dissolved in deionized water (2 mg/mL; pH 4) and spread using a ultrasound (Q-Sonica, Newtown, CT, USA) fixed with a tip (19 mm) [49]. Using a method previously specified, sonication was performed in an ice bath at 32 W of acoustic delivery power for 15 min with 8 s (pulse mode on) and 2 s (pulse mode off) [50].

To avoid particle deagglomeration, Ti particles was dispersed in high glucose DMEM enriched with 10% FBS (Gibco).

### 3.4. MTT Assay

The impacts of TiO_2_ NPs on the growth of Hep3 and Hep-G2 cells was measured using the MTT test and the cell lines were obtained from NCCS, Pune, India. Both cells were cultivated separately in a 96-well plate and treated with the TiO_2_ NPs at various doses (3.13, 6.25, 12.5, 25, 50, 100, and 200 µg/mL) for 24 h. The viability of control and treated Hep3 and Hep-G2 cells were assessed using the previous method [51].

### 3.5. Dual Staining

TiO_2_ NPs induced apoptosis in Hep3 and Hep-G2 cells was studied by AO/EtBr staining. Both cells were cultivated separately on a 24-well plate. They were administered with the IC_25_ (low dose) and IC_50_ (high dose) concentrations of TiO_2_ NPs and standard drug 5-Fluorouracil (5-FU, 10 µM) for 24 h. Later, both cells were stained using 100 µg/mL of AO/EtBr for 5 min. Under a fluorescent microscope, the produced fluorescence was examined to detect apoptosis [52].

### 3.6. Comet Assay

DNA damage was studied using a comet assay, which has been discussed earlier. All tests were conducted at 4 °C in red light. 24 h after seeding, a monolayer of Hep3 and HepG2 cells was employed to collect cells for the assay. Cells were trypsinized at 12 and 24 h after treatment with IC25 and IC50 doses of TiO_2_ NPs and the reference drug 5-Fluorouracil (5-FU, 10 µM). A 1% solution of normal agarose in PBS was carefully poured over a completely frozen micro slide, sealed with a coverslip, and cooled for about 5 min in an ice bath. Once the gel had solidified, the coverslip was taken. At 37 °C, a 1:3 ratio of cell suspension was added with low melting agarose (1%). After the gel had set, the coating mixture (100 µL) was promptly placed on top, spread evenly around the micro slide, and left to solidify. After letting the gel containing the cell solution settle, a third coating of 100 µL agarose (1%) was added. Once the agarose had set, the coverslips was detached and the slides were placed in a container containing ice-cold lysis buffer (2.5 µM NaCl, 100 mM Na2EDTA, 10 mM Tris, NaOH: pH 10, 0.1% Triton X-100) and cooled at 4 °C for 16 h. To prevent DNA damage, the aforementioned procedures were executed with minimal light. They were taken out of the lysis solution and laid down on the bottom of an electrophoresis tank. Electrophoresis buffer (300 mM NaOH, 1 mM Na2EDTA, pH > 13) was used to fill the reservoirs to the point where the slides could be submerged. After 20 min of incubation in the buffer (to facilitate DNA unwinding), electrophoresis was executed at 0.8 V/cm for 15 min. The slides were withdrawn from the electrophoresis, rinsed thrice in a neutralization buffer (0.4 M Tris, pH 7.5), and then tapped carefully to dry. EtBr (50 µg/mL) was used to stain nuclear DNA in a volume of 20 µL. The Carl Zeiss epifluorescence microscope was used to capture the images. Image analysis software (CASP software) was employed to examine digital images of 200 cells from each treatment. DNA amount of specific nuclei was calculated, and the extent of DNA injury was assessed as a percentage of total DNA in the tail [53] using the photographs.

### 3.7. DAPI Staining

The apoptotic nuclear morphological changes induced by TiO_2_ NPs in Hep3 and Hep-G2 cells were analyzed using DAPI staining. After seeding both cells separately into a 24-well plate, cells were exposed to the IC_25_ (low dose) and IC_50_ (high dose) doses of TiO_2_ NPs and standard drug 5-FU (10 µM) for 24 h. Then cells were stained using DAPI (200 µg/mL) for 15 min. Finally, a fluorescence microscope was employed to detect any apoptotic nuclear modifications [54].

### 3.8. Rhodamine-123 Staining

Rh-123 staining was executed to evaluate the MMP status of control and treated Hep3 and Hep-G2 cells. Briefly, cells were cultivated on a 24-well plate treated with and IC_25_ (low dose) and IC_50_ (high dose) concentrations of TiO_2_ NPs and the standard drug 5-FU (10 µM) for 24 h. Then 10 µg/mL of Rh-123 was loaded, and MMP was analyzed using a fluorescence microscope [55].

### 3.9. Quantification of Oxidative Stress Markers

The corresponding assay kits (Thermo Fisher Scientific, Waltham, MA, USA) were used to evaluate the antioxidants and oxidative stress markers such as MDA, SOD, CAT, and GSH in control and exposed to IC_25_ (low dose) and IC_50_ (high dose) concentrations of TiO_2_ NPs and standard drug 5-FU (10 µM) in Hep3 and Hep-G2 cells for 48 h. All the tests were performed in triplicate using the corresponding kits [56].

### 3.10. Measurement of Apoptotic Protein Levels

By using the respective assay kits, the apoptotic proteins (Bax, Bcl-2, CytC, p53, caspase-3, -8, and -9) in control and IC_25_ (low dose) and IC_50_ (high dose) doses of TiO_2_ NPs and standard drug 5-FU (10 µM) treated Hep3 and Hep-G2 cells were assayed using the recommended instructions of the manufacturer (Thermo Fisher, USA) [57].

### 3.11. Statistical Methods

The mean ± SD are utilized to express the values. One-way ANOVA was executed to examine the values, and Duncan’s multiple range test for post hoc evaluation. For data analysis, SPSS V 17.0 software was used. For all tests, a significance was specified as *p* < 0.05.

## 4. Conclusions

In conclusion, TiO_2_ NPs were produced via a green route using *Syzygium cumini* seed aqueous extract. The XRD patterns show synthesized TiO_2_ NPs exhibit a rutile structure and an average crystalline size of 55 nm. The photoluminescence spectra of TiO_2_ NPs show green emissions at 530 nm. The antimicrobial effect of TiO_2_ was likewise similar to the standard drug amoxicillin. Furthermore, the formulated TiO_2_ NPs effectively inhibited the viability of Hep3 and Hep-G2 cells. TiO_2_ NPs treatment considerably promoted ROS generation, reduced the MMP level, and induced apoptosis in both Hep3 and Hep-G2 cells. The decreased level of antioxidants and increased expressions of apoptotic proteins were also observed in the TiO_2_ NPs-treated Hep3 and Hep-G2 cells. Therefore, it was clear that TiO_2_ NPs will be helpful in biomedical applications, including treating infectious diseases as well as liver cancer.

## Data Availability

All the data related to this study are available from the corresponding author based upon request.
